# The OptiMUM-study: EMDR therapy in pregnant women with posttraumatic stress disorder after previous childbirth and pregnant women with fear of childbirth: design of a multicentre randomized controlled trial

**DOI:** 10.1080/20008198.2017.1293315

**Published:** 2017-02-24

**Authors:** M. A. M. Baas, C. A. I. Stramrood, L. M. Dijksman, A. de Jongh, M. G. van Pampus

**Affiliations:** ^a^ Department of Obstetrics and Gynaecology, Onze Lieve Vrouwe Gasthuis, Amsterdam, the Netherlands; ^b^ Department of Obstetrics and Gynaecology, University Medical Centre Utrecht, Utrecht, the Netherlands; ^c^ Department of Epidemiology, Onze Lieve Vrouwe Gasthuis, Amsterdam, the Netherlands; ^d^ Department of Quality and Safety, St. Antoniusziekenhuis, Nieuwegein, the Netherlands; ^e^ Department of Behavioral Sciences, Academic Centre for Dentistry Amsterdam (ACTA), Amsterdam, the Netherlands

**Keywords:** Posttraumatic stress disorder, fear of childbirth, eye movement desensitization and reprocessing, EMDR, PTSD, trauma, anxiety, pregnancy, postpartum, obstetrics

## Abstract

**Background**: Approximately 3% of women develop posttraumatic stress disorder (PTSD) after giving birth, and 7.5% of pregnant women show a pathological fear of childbirth (FoC). FoC or childbirth-related PTSD during (a subsequent) pregnancy can lead to a request for an elective caesarean section as well as adverse obstetrical and neonatal outcomes. For PTSD in general, and several subtypes of specific phobia, eye movement desensitization and reprocessing (EMDR) therapy has been proven effective, but little is known about the effects of applying EMDR during pregnancy.

**Objective**: To describe the protocol of the OptiMUM-study. The main aim of the study is to determine whether EMDR therapy is an effective and safe treatment for pregnant women with childbirth-related PTSD or FoC. In addition, the cost-effectiveness of this approach will be analysed.

**Method**: The single-blind OptiMUM-study consists of two two-armed randomized controlled trials (RCTs) with overlapping design. In several hospitals and community midwifery practices in Amsterdam, the Netherlands, all eligible pregnant women with a gestational age between eight and 20 weeks will be administered the Wijma delivery expectations questionnaire (WDEQ) to asses FoC. Multiparous women will also receive the PTSD checklist for DSM-5 (PCL-5) to screen for possible PTSD. The clinician administered PTSD scale (CAPS-5) will be used for assessing PTSD according to DSM-5 in women scoring above the PCL-5 cut-off value. Fifty women with childbirth-related PTSD and 120 women with FoC will be randomly allocated to either EMDR therapy carried out by a psychologist or care-as-usual. Women currently undergoing psychological treatment or women younger than 18 years will not be included. Primary outcome measures are severity of childbirth-related PTSD or FoC symptoms. Secondary outcomes are percentage of PTSD diagnoses, percentage caesarean sections, subjective childbirth experience, obstetrical and neonatal complications, and health care costs.

**Results**: The results are meant to provide more insight about the safety and possible effectiveness of EMDR therapy during pregnancy for women with PTSD or FoC.

**Conclusion**: This study is the first RCT studying efficacy and safety of EMDR in pregnant women with PTSD after childbirth or Fear of Childbirth.

## 1. Posttraumatic stress disorder after childbirth, and fear of childbirth

Although pregnancy and childbirth are supposed to be joyful times, 25% of pregnant women report having psychological problems (Vesga-López et al., 2008). Research indicates that up to 43% of women experience childbirth as traumatic (Alcorn, O’Donovan, Patrick, Creedy, & Devilly, 2010), and it is estimated that 3% of women will develop posttraumatic stress disorder (PTSD) following childbirth (Grekin & O’Hara, 2014). The main symptoms of PTSD, according to DSM-5, are re-experiencing, avoidance and numbing, negative cognitions and mood, and hyperarousal. These symptoms last more than one month and result in significant dysfunction (American Psychiatric Association, 2013). By definition, PTSD after previous childbirth refers to PTSD with childbirth itself being the index trauma. The distinction with non-childbirth related PTSD that is ongoing or retriggered in the postpartum period is not always made correctly (Grekin & O’Hara, 2014). PTSD following other traumatic events is beyond the scope of this study; however, a history of trauma or psychiatric disorders such as PTSD are risk factors for childbirth-related PTSD. Childbirth-related PTSD can occur in the absence of medical complications, but prevalence has been found to be higher among women with complicated pregnancies (Ayers, Bond, Bertullies, & Wijma, 2016). For example, it appears that 14% of women with preterm birth due to preeclampsia or premature preterm rupture of membranes develop PTSD (Stramrood et al., 2011). Posttraumatic stress symptoms after childbirth are not always self-limiting, leading to a chronic disorder (Söderquist, Wijma, & Wijma, 2006). Because the prospect of giving birth may trigger both memories of a previous distressing delivery as well as traumatic events such as sexual violence or previous medical trauma, pregnancy can be accompanied by severe childbirth-related anxiety and a disproportional fear of the upcoming delivery. In addition, avoidance symptoms of PTSD often manifest themselves as avoiding future pregnancy, avoiding prenatal care in a subsequent pregnancy, or demanding an elective caesarean section (Fuglenes, Aas, Botten, Øian, & Kristiansen, 2011; Gottvall & Waldenström, 2002). Moreover, (psychotraumatic) stress during pregnancy appears to be related to negative outcomes for the mother and the foetus (Alder, Fink, Bitzer, Hösli, & Holzgreve, 2007; Seng, Low, Sperlich, Ronis, & Liberzon, 2011; Seng et al., 2001), such as preterm birth (Shaw et al., 2014; Yonkers et al., 2014).

Women who are pregnant for the first time may also experience anxiety symptoms during pregnancy. Overall, about 7.5% of pregnant women experience a pathological fear of childbirth (FoC) (Adams, Eberhard-Gran, & Eskild, 2012; Söderquist, Wijma, Thorbert, & Wijma, 2009). FoC has been found in both nulliparous women who have not experienced childbirth before (primary FoC), as well as in multiparous women (women with a previous birth, whether one or more) in whom a negative or traumatic previous childbirth experience often plays a role (secondary FoC) (Hofberg & Ward, 2003; Størksen, Garthus-Niegel, Vangen, & Eberhard-Gran, 2013). Therefore, it is to be expected that (subclinical) PTSD is accompanied by FoC. FoC appears to be more common in nulliparous women compared to multiparous women (Rouhe, Salmela-Aro, Halmesmäki, & Saisto, 2009). Several studies found that the concept of FoC is multifaceted, including – but not limited to – fear of pain, the baby dying or being handicapped, loneliness, lack of support, or being concerned, or embarrassed about, one’s own appearance while giving birth (Garthus-Niegel, Størksen, Torgersen, Von Soest, & Eberhard-Gran, 2011; Huizink, Mulder, Robies De Medina, Visser, & Buitelaar, 2004). Women with FoC may attempt to avoid pregnancy or even decide to terminate the pregnancy, or request (and receive) a caesarean section (Raïsanen et al., 2014; Størksen, Garthus-Niegel, Adams, Vangen, & Eberhard-Gran, 2015). Moreover, FoC has been found to be associated with increased risk of emergency caesarean section (Ryding, Wijma, Wijma, & Rydhström, 1998), and a sixfold increased risk of PTSD following childbirth (Söderquist et al., 2009).

Prevalence rates of women avoiding pregnancy because of posttraumatic stress symptoms after previous childbirth or FoC are unknown. Avoidance symptoms can include avoiding conversations with healthcare providers about childbirth-related mental health problems, such that even women with the most severe symptoms might not be noticed. Awareness among healthcare providers and women themselves, and an available effective treatment may help to improve quality of life for them and their partners.

## 2. Therapeutic attitudes and practice

### 2.1. Fear of childbirth

Related to FoC, a small number of intervention studies have been published, of which a few are controlled trials, and only three were randomized controlled trials (RCT) (Rouhe et al., 2015a; Saisto, Salmela-Aro, Nurmi, Könönen, & Halmesmäki, 2001; Toohill et al., 2014). In 2001, 176 pregnant women were randomized between conventional and intensive therapy (Saisto et al., 2001). Conventional therapy included routine obstetric appointments, standard distribution of written information about pain relief and pros and cons of vaginal delivery versus caesarean section. In the intensive therapy group, appointments were combined with cognitive therapy, and additive informative appointments were recommended. In the intensive therapy group, birth concerns and anxiety were more reduced compared to the conventional group, and labour duration was shorter (mean ± standard deviation; 6.8 ± 3.8 h, compared to the conventional group 8.5 ± 4.8 h, *p* *= *0.04). In both groups the amount of requests for elective caesarean was decreased with no difference between the groups. In a randomized controlled trial among nulliparae with severe FoC, participating in group psycho-education in combination with relaxation, exercises resulted in a more positive childbirth experience (36.1% versus 22.8%, *p* = 0.04), and decreased postpartum depressive symptoms (mean sum score 6.4 ± 5.4 versus 8.0 ± 5.9, *p* = 0.04) (Rouhe et al., 2013, 2015a). The number of uncomplicated vaginal deliveries was significantly higher among women in the intervention group than among the controls (63.4% versus 47.5%, *p* < 0.01), who received care by community nurses and were referred if considered necessary. Because complications lead to more medical costs, a greater number of uncomplicated deliveries reduced medical costs in the intervention group, resulting in group psycho-education being cost-neutral (Rouhe et al., 2015b). In another RCT a telephone psycho-education counselling intervention offered by midwives for 189 pregnant women with FoC was found to be significantly more effective regarding the reduction of childbirth fear levels than care-as-usual (CAU) (clinically meaningful improvement in FoC in 49% versus 26%, *p* = 0.002) (Toohill et al., 2014). A non-randomized controlled trial among 106 pregnant women showed that women who received counselling for FoC by a team of specialized midwives were satisfied with the counselling they received during their pregnancy (Ryding, Persson, Onell, & Kvist, 2003). However, postpartum these women also indicated having had significantly more negative delivery experience compared to the control group (44.3 ± 20.5 versus 29.7 ± 17.4, *p* < 0.01). They also reported posttraumatic stress symptoms more frequently than a matched sample of women from the general parturient population (19% versus 2%). In conclusion, treatment of FoC can lead to a reduction in anxiety, shorter labour duration, and a greater likelihood of uncomplicated vaginal childbirth. Results about the impact of treatment of FoC on childbirth experience are contradictory, and negative effects on posttraumatic stress symptoms have been described (Ryding et al., 2003). However, the amount of previous results, and in specific RCTs, is scarce.

A disproportionate FoC with a level of symptoms meeting the DSM-5 criteria for specific phobia is often referred to as tocophobia (American Psychiatric Association, 2013). Cognitive behavioural therapy, particularly *in vivo* exposure, is generally considered to be the treatment of choice for specific phobias (Barlow, Allen, & Basden, 2007; Wolitzky-Taylor, Horowitz, Powers, & Telch, 2008). However, because *in vivo* exposure is hardly possible for women with FoC, and the fact that EMDR therapy has been found to be effective for a wide variety of specific phobia subtypes (de Jongh, Holmshaw, Carswell, & Van Wijk, 2011; de Jongh, Ten Broeke, & Renssen, 1999; Doering, Ohlmeier, de Jongh, Hofmann, & Bisping, 2013), this may be a promising alternative. Thus far, controlled studies using EMDR therapy among pregnant women with FoC are lacking.

### 2.2. Posttraumatic stress disorder after childbirth

Since EMDR therapy can be used to treat PTSD resulting from a wide range of traumatic events, it is unlikely that the effectiveness would differ when the traumatic event is childbirth. There are some indications that EMDR therapy is experienced as less intensive than prolonged exposure therapy by the patients, with patients being distracted during therapy; and that there is a faster reduction in symptoms compared to other treatments (Ho & Lee, 2012; Nijdam, Gersons, Reitsma, de Jongh, & Olff, 2012). However, research about treating PTSD following childbirth is limited to some small uncontrolled trials (Lapp, Agbokou, Peretti, & Ferreri, 2010), and even less is known about the benefits and risks of treatment during subsequent pregnancy. Only two small, uncontrolled studies with respectively four women (of which one pregnant) (Sandström, Wiberg, Wikman, Willman, & Högberg, 2008), and three pregnant women (Stramrood et al., 2012) provide some preliminary support for the notion that EMDR therapy can be safe and effective for PTSD following childbirth. A pilot study with four women (of which one pregnant), who fulfilled DSM-IV criteria for PTSD, received an unknown number of EMDR therapy sessions (Sandström et al., 2008). Posttraumatic stress symptoms were reduced (mean score TES 52.7 before, 33.5 after treatment, 22.7 at follow-up), and two participants who completed the follow-up measurements no longer fulfilled the DSM-IV criteria for PTSD after treatment. In a case series three pregnant women with PTSD-symptoms received one or two EMDR therapy sessions followed by a closing session or another three sessions of EMDR therapy for different distressing events (Stramrood et al., 2012). It was found that despite several medical complications, their PTSD symptoms decreased, confidence in the upcoming delivery improved, and that they evaluated their subsequent delivery experience positively. In conclusion, preliminary results show that treatment of childbirth-related PTSD during subsequent pregnancy using EMDR can lead to a reduction in symptoms. Randomized research with validated questionnaires is needed.

Yet, it should be noted that the fact that patients are still continuously confronted with the prospect of the upcoming and inevitable delivery might complicate the condition and treatment. This could easily lead to clinicians being reluctant to offer any treatment at all. In addition, clinicians might be afraid that trauma-focused therapy during pregnancy could provoke a stress response with negative effects on the mother or the foetus. On the other hand, it could be argued that untreated PTSD results in a continuously overactive stress system, which may lead to complications during pregnancy. Furthermore, PTSD is known to have a potentially negative influence on mother–child bonding and the relationship of the mother with her partner (Ayers, Eagle, & Waring, 2006).

## 3. Objectives

Our main objective is to assess the safety and efficacy of EMDR therapy for pregnant women with PTSD after previous childbirth or FoC. The following hypotheses are tested:

### 3.1. Posttraumatic stress disorder

Within the treatment group we expect a significant reduction of the severity of PTSD symptoms and percentage of PTSD diagnoses present following EMDR therapy. We expect a significantly larger reduction in PTSD symptom severity and a significantly lower percentage of PTSD diagnoses in the treatment group after completion of the EMDR sessions, compared to the CAU group. Furthermore, we predict a significantly lower percentage of caesarean sections (both elective and unplanned), a significantly more positive childbirth experience, and significantly lower health care costs in the treatment group.

### 3.2. Fear of childbirth

Within the treatment group we expect a significant reduction in FoC symptoms following EMDR therapy, a significantly larger reduction in FoC symptoms, a significantly lower percentage of caesarean sections (elective and unplanned), lower health care costs, and a more positive childbirth experience in the treatment group, as compared to the CAU group.

Not least importantly, in both arms we do not predict EMDR therapy to lead to significantly more obstetric or neonatal complications than CAU.

## 4. Design/methods

### 4.1. Participants

Participants eligible for screening for the OptiMUM-study are women with a gestational age of 8–20 weeks, who master the Dutch language (written and spoken). Exclusion criteria are age <18 years old, current psychological treatment, intermediate or high suicide risk (based on the mini international neuropsychiatric interview-plus; MINI-plus), or severe psychotic disorder, such as schizophrenia or current psychosis (based on MINI-plus). Participants are recruited from one university hospital, two teaching hospitals and several community midwifery practices in Amsterdam, the Netherlands..

### 4.2. Design

**Figure 1.**
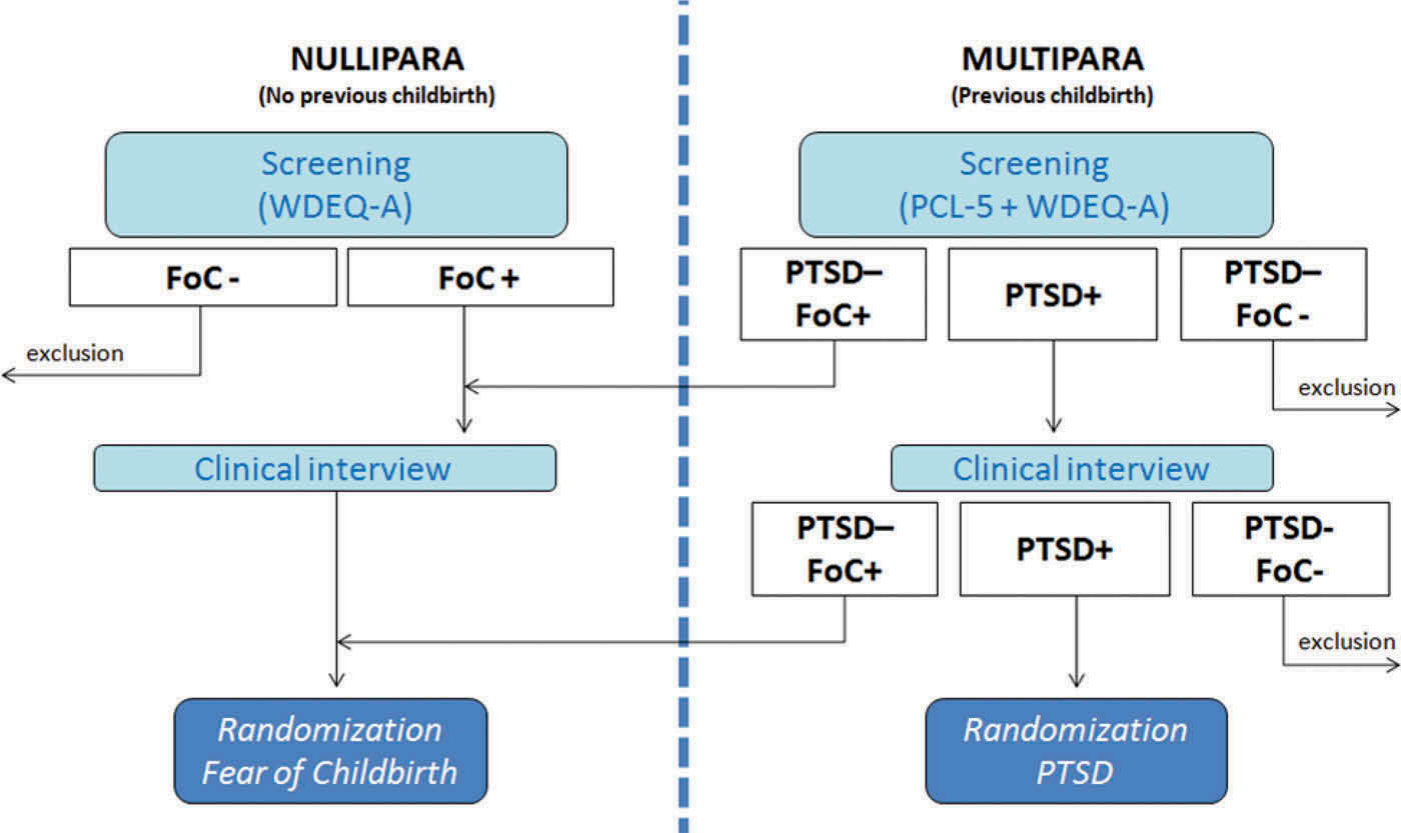
Procedure of inclusion. WDEQ-A: Wijma delivery expectations/experience questionnaire; PCL-5: PTSD checklist for DSM-5; FoC: fear of childbirth; PTSD: posttraumatic stress disorder.

Patients will be randomized to either the EMDR therapy group or CAU group on a 1:1 basis by block randomization. The randomization is done by an independent computer program. FoC and PTSD after childbirth have their own randomization program, and therefore can be seen as two separate randomized controlled trials with overlapping design (see Figure 1). The randomization procedure will not balance sites, and no other stratification variables are used since there are no prognostics factors known regarding EMDR response among pregnant women. The study was approved by The Medical Research Ethics Committee of the OLVG Hospital, and is registered as NL4930410014 and NL4930510014. The study is registered prospectively in the Dutch trial register (www.trialregister.nl) as NTR5123 and NTR5122.

### 4.3. Intervention

Participants will be randomized to either EMDR therapy or CAU.

#### 4.3.1 EMDR therapy group (fear of childbirth: *n* = 60; PTSD: *n* = 25)

The EMDR therapy group will receive a maximum of three 90-min sessions, in addition to standard care during pregnancy. EMDR is a psychological intervention that has been developed for the treatment of traumatic memories (Oren & Solomon, 2012). It is internationally recognized as a first choice therapy for treating PTSD (NICE, 2005; WHO, 2013). EMDR therapy will be applied according to the Dutch translation of the standard EMDR protocol. The standardized procedure involves a combination of (1) focusing on the most distressing images of the traumatic event (or in case of FoC: worst-case scenario), including corresponding emotions, cognitions and physical response; and (2) repeated series of eye movements as a bilateral stimulation, followed by free associations. Current distress is rated until the distress level is (as close to) zero, after which a positive cognition is introduced corresponding to the image. The hypothesis is the traumatic event is unprocessed. By taxing patient’s working memory by both the image and eye movements simultaneously, the memory is reconsolidated less vividly. All therapy sessions will be videotaped. Tapes will be scored to determine treatment fidelity by using the EMDR Fidelity Scale (Korn, Zangwill, Lipke, & Smyth, 2001).

#### 4.3.2 Care-as-usual group (fear of childbirth: *n* = 60; PTSD: n = 25)

CAU is defined as standard care during pregnancy, with routine obstetrical checks. Assuming good clinical care, anxious pregnant women and those with traumatic childbirth experiences may receive more counselling compared to not-anxious pregnant women, but will (probably) not be referred for EMDR therapy. Type and frequency of any form of professional care will be registered.

### 4.4. Procedure

**Figure 2.**
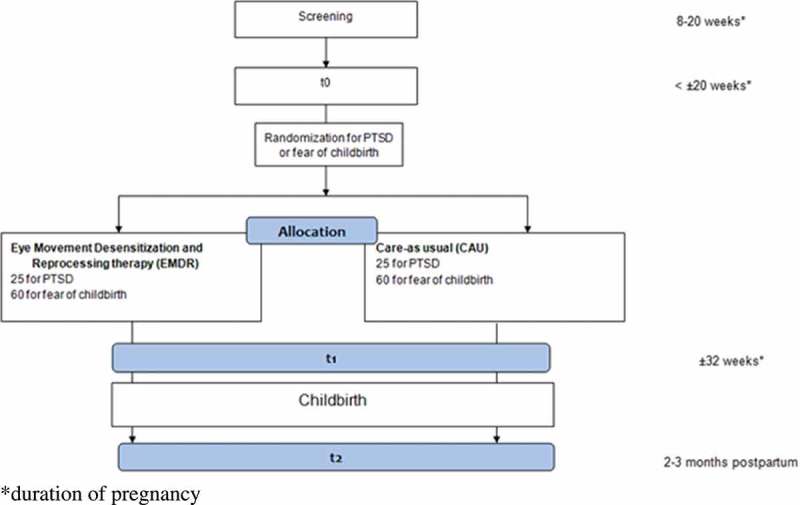
Study design.

Women with a gestational age between eight and 20 weeks, regardless of parity, who score above the cut-off score of the Wijma delivery expectations questionnaire (WDEQ-A) at time of screening will be invited for a clinical interview (see Figure 2). In case of multiparity, the PTSD checklist for DSM-5 (PCL-5) is administered at time of screening in addition.

During the clinical interview, traumatic events are explored in all women with the life events checklist (LEC-5). If appropriate, subsequently the clinician administered PTSD scale (CAPS) will be administered to diagnose PTSD (childbirth related, or not). In case of several traumatic events, the CAPS will be administered for each event separately. A pregnant woman is eligible for randomization for PTSD when a PTSD diagnosis can be obtained using the CAPS, with previous childbirth being the index trauma. PTSD with index trauma other than childbirth will be registered and analysed as co-existing psychiatric disorder. When informed consent is obtained, computer-assisted randomization will take place to allocate participants to either EMDR therapy or CAU. If the woman does not meet the criteria for PTSD after previous childbirth, but does score above cut-off on the WDEQ-A, she is eligible to be randomized for FoC (Figure 1). Measurements of (PTSD and/or FoC) symptoms take place between sessions (with childbirth being the index event of the PSS-SR questionnaire): at the start of each therapy session, or in case of CAU, measurements are done two, four and six weeks after randomization by email. Post-treatment antepartum measurement takes place at about 32–34 weeks of gestational age by a blinded assessor. If there is unblinding during the interview, another assessor will finish the interview. Final follow-up takes place two to three months after childbirth. Perinatal healthcare providers are semi-blinded: randomization results are not sent to the professionals, but if a pregnant woman would like to share information with her healthcare provider it is not prohibited.

A limitation for recruitment until 20 weeks of gestation has been chosen to ensure that there is enough time for planning an interview, arranging for therapy logistics, and for scheduling three therapy sessions before post-treatment sessions will take place around 32–34 weeks of gestation.

### 4.5. Instruments

The order in which the instruments are administered can be seen in Table 1. The following instruments are used:

**Table 1.** Timing of instruments.

			In between sessions		t1		t2	
	Screening	t0	EMDR	CAU	EMDR	CAU	EMDR	CAU
Instruments								
*Clinical interview*								
CAPS(+LEC-5)	-	x*	-	-	x*	x *	-	-
MINI-plus	-	x	-	-	x	x	-	-
*Self-report questionnaires*								
PCL-5	x	-	-	-	-	-	-	-
PSS-SR*	-	x	x	x	x	x	x	x
HADS	-	x	-	-	x	x	x	x
Satisfaction scale	-	-	-	-	-	-	x	x
WDEQ								
version A	x	-	x	-	x	x	-	-
version B	-	-	-	-	-	-	x	x

CAPS: clinician-administered PTSD scale; CAU: care-as-usual; EMDR: eye movement desensitization and reprocessing therapy; HADS: hospital anxiety and depression scale; LEC-5: life events checklist; MINI-plus: mini international neuropsychiatric interview-plus; PCL-5: PTSD checklist for DSM-5; PSS-SR: PTSD symptom scale; PTSD: posttraumatic stress disorder; WDEQ-A/B: Wijma delivery expectations/experiences questionnaire

*Only if experienced a traumatic event.

#### 4.5.1 PTSD checklist for DSM-5 (PCL-5) (Boeschoten, Bakker, Jongedijk, & Olff, 2014; Weathers et al., 2013)

The PCL-5 is the DSM-5 version of the PCL. The PCL-5 is a 20-item self-report questionnaire assessing the 20 symptoms of PTSD according to DSM-5. Participants are asked to rate the severity of PTSD symptoms in the past month on a five-point scale from 0 (‘not at all’) to 4 (‘extremely’). Women meet the cut-off value when they report symptoms in accordance with the DSM-5 criteria: each item rated 2 (‘moderately’) or higher counts as a symptom, in addition at least one B item (questions 1–5), one C item (questions 6–7), two D items (questions 8–14) and two E items (questions 15–20) are required to meet the cut-off. Initial psychometric evaluation showed a strong internal consistency (0.94) (Blevins, Weathers, Davis, Witte, & Domino, 2015).

#### 4.5.2 PTSD symptom scale (PSS-SR) (Foa, Cashman, Jaycox, & Perry, 1997)

The PSS-SR is a 17-item self-report version of the semi-structured questionnaire to assess symptoms of PTSD in the past week. Items are scored on a four-point scale from 0 (‘not at all’/‘only one time’) to 3(‘almost always’/‘five or more times a week’). Psychometric properties are good, with internal consistency and test-retest reliability being 0.91 and 0.74, respectively (Foa et al., 1997).

#### 4.5.3 Life events checklist (LEC-5) (Weathers et al., 2013)

The LEC-5 is a self-report questionnaire to screen for potentially traumatic events during one’s life. Sixteen events which are known for their potential to cause PTSD, and one additional item for other extreme events are assessed. In our study it is used to identify the index traumatic event for administration of the CAPS-5.

#### 4.5.4 Clinician-administered PTSD scale (CAPS-5) (Boeschoten et al., 2014)

The CAPS-5 is the gold standard for diagnosing PTSD. It is a structured interview comprising 30 items including all 20 DSM-5 PTSD symptoms. Per item a severity rating is calculated by combining information about frequency and intensity. For DSM-5 PTSD diagnosis, a symptom is considered present when severity score is rated 2 (‘moderate’) or higher (range 0–4). Following DSM-5 criteria, one B, one C, two D and two E criteria are required. Research on the psychometric properties of the Dutch translation of CAPS-5 is currently being conducted, but the internal consistency of the Dutch CAPS-IV was good, ranging from 0.63 to 0.79 for clusters, to 0.89 overall (Hovens et al., 1994).

#### 4.5.5 Mini international neuropsychiatric interview-plus (MINI-plus) (Sheehan et al., 1998)

The MINI-plus is a structured interview for axis I DSM-IV conditions. In the current study, it is used to evaluate comorbidity, among which are mood disorders (i.e. major depressive episode, manic episode), anxiety disorders (i.e. panic disorder, specific phobia), substance abuse (alcohol, drugs), and psychotic disorders. Each chapter starts with one or more screening questions, and, if positive, is followed by further exploration before a specific diagnosis is made. There is a reasonable to good concurrent validity.

#### 4.5.6 Hospital anxiety and depression scale (HADS) (Zigmond & Snaith, 1983)

The HADS is a 14-item self-report questionnaire with a two-factor structure to measure anxiety and depressive symptoms. Each item is scored 0–3. Each subscale had a cut-off level of ≥8 for clinical significance. Internal consistency of the total scale and both subscales is found to be good (range 0.71–0.9) (Spinhoven et al., 1997).

#### 4.5.7 Satisfaction scale

Satisfaction around pregnancy and delivery is measured by the question ‘how satisfied are you about your pregnancy’, and ‘how satisfied are you about your delivery’ on a 10-point scale, where 0 equals extremely unsatisfied, and 10 extremely satisfied.

#### 4.5.8 Wijma delivery expectations/experiences questionnaire (WDEQ-A/B) (Wijma, Wijma, & Zar, 1998)

The WDEQ is a 33-item self-report questionnaire assessing FoC during pregnancy (version A), and after delivery (version B) in terms of the woman’s cognitive appraisal of childbirth. It was designed as a monofactorial scale, all 33 items being scored on a six-point scale leading to a sum score between 0 and 165, with higher score equalling more FoC. The WDEQ can be dichotomized to conclude if there is FoC or not (cut-off ≥85). Internal consistency is found to be high (≥0.87) (Wijma et al., 1998).

### 4.6. Interrater reliability and treatment fidelity

All clinical interviewers receive an official training in assessment of the MINI and CAPS. Interrater reliability of assessment of the clinical interviews (MINI, CAPS) will be measured by scoring interviews by two independent assessors, or group wise scoring video tapes. Interrater reliability will be enhanced by supervision of each clinical interview.

All four therapists are psychologists with a postdoctoral degree in psychology. They are not part of the research team, and work at different sites. They have completed the basic and advanced EMDR training course accredited by the Dutch National EMDR Association (www.emdr.nl), and have at least one year experience with providing EMDR therapy. Patients will be randomly and equally distributed among therapists. All therapy sessions will be videotaped, and a selection will be rated for treatment fidelity. Group supervision every three to four months is obligatory for all therapists during the whole study duration. After each first session with a new patient, a case conceptualization will be emailed for supervision, including: the story of the traumatic event, with a hierarchy of the most relevant traumatic moments (targets) regarding to the current impairment. Deviations from protocol will be noted and reported.

The reliability of medical record data will be ascertained by double data entry of 20% of all records, and there is a build-in quality check in the data entry system.

### 4.7. Sample size

The main treatment outcomes of the current study are the effectiveness of EMDR therapy for treating PTSD and FoC. In practice, this corresponds to the difference in total CAPS or WDEQ scores between the EMDR and CAU group at follow-up during pregnancy. In both cases, a significance level of 0.05 and power of 80% is used. A one-sided significance level is chosen according with our hypothesis to find a reduction in PTSD or FoC symptoms. For PTSD, expecting a large effect size of at least 0.8 (Gerger et al., 2014), this results in 21 patients needed per group. The sample size was set at 25 patients each group, calculating for 20% patient attrition. For FoC, EMDR therapy effect size is not known yet. The most comparable data is EMDR therapy for dental phobia, which showed a large effect size (Doering et al., 2013). We estimated a medium effect size of 0.5, which results in 50 patients needed per group. With 20% patient attrition, this leads to a requirement of two groups of 60 patients for FoC.

### 4.8. Statistical analyses

Descriptive statistics will be used to evaluate demographic, clinical (obstetrical and psychological) baseline characteristics of both arms. All data will be analysed according to intention to treat analysis: the data of all randomized subjects will be analysed using the groups (treatment vs. CAU) as defined at randomization. In both conditions symptoms will be measured at regular intervals, in order to monitor treatment results over time. The treatment group and CAU group will be compared on all outcomes, which will be analysed with linear mixed models (LMM). Baseline scores will be included as covariates, time as a categorical variable, and treatment condition as a fixed effect. The intercept will be treated as a random effect. Furthermore, we will use Chi-squared tests and independent *t*-tests to compare both treatment conditions at different times (alpha level 0.05), or Fisher’s Exact test and Mann–Whitney U-test when appropriate. Effect sizes from change in symptoms between baseline and post-treatment antepartum, and post-treatment postpartum will be calculated with Cohen’s d. Safety is objectified by measuring worsening of FoC/PTSD symptoms, neonatal and obstetrical outcomes, dropout, and determining the presence of adverse events. However, our study is not powered upon these safety measures, therefore this outcome will be shown descriptively.

All analyses will be performed using SPSS version 22.0 (IBM Corp, 2013).

### 4.9. Cost-effectiveness analysis

A cost-effectiveness analysis by a societal perspective will be performed, including a sensitivity analysis. All costs in maternal care during pregnancy, delivery and the first three months postpartum will be collected. This includes direct costs (e.g. visits to primary healthcare and obstetric hospital services, procedures, medication, and maternal admission), but also indirect costs (e.g. prolonged maternity leave, partner taking days off). Also, self-reported costs made by the patient will be collected (e.g. alternative medicine, antenatal courses). In addition, neonatal admission costs will be included. As far as possible, true costs will be collected besides using the Dutch system of hospital pricing that is based on diagnosis and procedure codes. Information will be derived from obstetric patient records, and the postpartum questionnaire that includes questions about healthcare use and additional costs.

## 5. Discussion

The OptiMUM-study has several strengths resulting in a potentially innovative study. Firstly, it is a randomized controlled trial which will provide answers in a patient population where research is scarce. Secondly, the present study is a large study, comprising inclusion in university and teaching hospitals, but also in community midwifery practices. This will result in a decent cross-section of the population and including women with low, medium and high risk pregnancies. Also, PTSD and psychological comorbidity will be assessed with validated clinical interviews, which is an added value compared to many previous studies using self-report questionnaires (Rouhe et al., 2015a; Saisto et al., 2001; Toohill et al., 2014). Furthermore, besides effectiveness, obstetric and neonatal outcome is taken into account. This may be an important contribution since our study is the first controlled study with an intervention for PTSD during pregnancy instead of postpartum. Finally, cost-effectiveness analysis is added to this study. As we expect symptoms of PTSD or FoC to decrease as a result of EMDR therapy, it is likely that costs of treatment are well compensated by prevented caesarean section and fewer complications; in particular because costs of EMDR therapy are relatively low compared to costs of medical complications.

There are several limitations to be expected. Comparing EMDR with CAU is a compromise, since there is no gold standard intervention during pregnancy. Adding a waitlist-control condition was not possible, since childbirth characteristics are the outcome measures in this study. The same holds true for a placebo-intervention group due to limited financial resources at the start of this study. We estimate the influence of more patient-professional contacts (6.5 h instead of 2 h) for the EMDR group as a small confounding factor. It is also possible that patients in the CAU group will search for professional help as a result of the screening procedure for this study, which potentially could raise awareness about their PTSD symptoms or FoC. Data about all types of treatments followed by patients (in both groups) are collected. Lastly, while prevalence rates of PTSD after childbirth are known, it is however unknown how many of those women will have a subsequent pregnancy.

If EMDR therapy proves to be safe and effective during pregnancy in treating PTSD after childbirth and FoC, these are strong arguments for standard screening pregnant women for these psychological conditions, and to refer them for treatment in an early phase of pregnancy.

## 6. Trial status

Currently screening and recruiting participants.

## Supplementary Material

Dutch Extended Abstract

## Highlights of the article

- For the 7.5% of the pregnant women showing a pathological FoC, and the 3% of women who develop PTSD after giving birth, pregnancy can be accompanied by severe psychological distress.
- The OptiMUM-study is the first controlled study with EMDR therapy for PTSD or FoC during pregnancy.
- In this trial, in addition to effectiveness, obstetric and neonatal outcomes are studied.

## References

[CIT0001] Adams S. S., Eberhard-Gran M., Eskild A. (2012). Fear of childbirth and duration of labour: A study of 2206 women with intended vaginal delivery. *BJOG: an International Journal of Obstetrics & Gynaecology*.

[CIT0002] Alcorn K. L., O’Donovan A., Patrick J. C., Creedy D., Devilly G. J. (2010). A prospective longitudinal study of the prevalence of post-traumatic stress disorder resulting from childbirth events. *Psychological Medicine*.

[CIT0003] Alder J., Fink N., Bitzer J., Hösli I., Holzgreve W. (2007). Depression and anxiety during pregnancy: A risk factor for obstetric, fetal and neonatal outcome? A critical review of the literature. *The Journal of Maternal-Fetal & Neonatal Medicine*.

[CIT0004] American Psychiatric Association (2013). *Diagnostic and statistical manual of mental disorders*.

[CIT0005] Ayers S., Bond R., Bertullies S., Wijma K. (2016). The aetiology of post-traumatic stress following childbirth: A meta-analysis and theoretical framework. *Psychological Medicine*.

[CIT0006] Ayers S., Eagle A., Waring H. (2006). The effects of childbirth related post-traumatic stress disorder on women and their relationships: A qualitative study. *Psychology, Health & Medicine*.

[CIT0007] Barlow D. H., Allen L. B., Basden S. L., Nathan P. E., Gorman J. M. (2007). Psychological treatments for panic disorders, phobias, and generalized anxiety disorder. *A guide to treatments that work*.

[CIT0008] Blevins C., Weathers F., Davis M., Witte T., Domino J. (2015). The posttraumatic stress disorder checklist for DSM-5 (PCL-5): Development and initial psychometric evaluation. *Journal of Traumatic Stress*.

[CIT0009] Boeschoten M. A., Bakker A., Jongedijk R. A., Olff M. (2014). *PTSD checklist for DSM-5– Dutch version*.

[CIT0010] Boeschoten M. A., Bakker A., Jongedijk R. A., Van Minnen A., Elzinga B. M., Rademaker A. R., Olff M. (2014). *Clinician administered PTSD scale for DSM-5 – Dutch version*.

[CIT0011] de Jongh A., Holmshaw M., Carswell W., Van Wijk A. (2011). Usefulness of a trauma-focused treatment approach for travel phobia. *Clinical Psychology & Psychotherapy*.

[CIT0012] de Jongh A., Ten Broeke E., Renssen M. R. (1999). Treatment of specific phobias with eye movement desensitization and reprocessing (EMDR): Protocol, empirical status, and conceptual issues. *Journal of Anxiety Disorders*.

[CIT0013] Doering S., Ohlmeier M. C., de Jongh A., Hofmann A., Bisping V. (2013). Efficacy of a trauma-focused treatment approach for dental phobia: A randomized clinical trial. *European Journal of Oral Sciences*.

[CIT0014] Foa E. B., Cashman L., Jaycox L., Perry K. (1997). The validation of a self-report measure of posttraumatic stress disorder: The Posttraumatic Diagnostic Scale. *Psychological Assessment*.

[CIT0015] Fuglenes D., Aas E., Botten G., Øian P., Kristiansen I. S. (2011). Why do some pregnant women prefer cesarean? The influence of parity, delivery experiences, and fear. *American Journal of Obstetrics & Gynecology*.

[CIT0016] Garthus-Niegel S., Størksen H. T., Torgersen L., Von Soest T., Eberhard-Gran M. (2011). The Wijma delivery expectancy/experience questionnaire: A factor analytic study. *Journal of Psychosomatic Obstetrics and Gynaecology*.

[CIT0017] Gerger H., Munder T., Gemperli A., Nüesch E., Trelle S., Jüni P., Barth J. (2014). Integrating fragmented evidence by network meta-analysis: Relative effectiveness of psychological interventions for adults with post-traumatic stress disorder. *Psychological Medicine*.

[CIT0018] Gottvall K., Waldenström U. (2002). Does a traumatic birth experience have an impact on future reproduction?. *British Journal of Obstetrics and Gynaecology*.

[CIT0019] Grekin R., O’Hara M. (2014). Prevalence and risk factors of postpartum posttraumatic stress disorder: A meta-analysis. *Clinical Psychology Review*.

[CIT0020] Ho M. S. K., Lee C. W. (2012). Cognitive behaviour therapy versus eye movement desensitization and reprocessing for post-traumatic disorder–is it all in the homework then?. *Revue Européenne De Psychologie Appliquée*.

[CIT0021] Hofberg K., Ward M. R. (2003). Fear of pregnancy and childbirth. *Postgraduate Medical Journal*.

[CIT0022] Hovens J., Van der Ploeg H., Klaarenbeek M., Bramsen I., Schreuder J., Rivero V. (1994). The assessment of posttraumatic stress disorder: With the clinician administered PTSD scale: Dutch results. *Journal of Clinical Psychology*.

[CIT0023] Huizink A. C., Mulder E. J., Robies de Medina P. G., Visser G. H., Buitelaar J. K. (2004). Is pregnancy anxiety a distinctive syndrome?. *Early Human Development*.

[CIT0024] IBM Corp (2013). IBM SPSS Statistics for Windows, Version 22.0.

[CIT0025] Korn D. L., Zangwill W., Lipke H., Smyth N. J. (2001). *EMDR fidelity scale*.

[CIT0026] Lapp L. K., Agbokou C., Peretti C. S., Ferreri F. (2010). Management of post traumatic stress disorder after childbirth: A review. *Journal of Psychosomatic Obstetrics and Gynaecology*.

[CIT0027] NICE (2005). *Post-traumatic stress disorder (PTSD): The treatment of PTSD in adults and children. Understanding NICE guidance*.

[CIT0028] Nijdam M. J., Gersons B. P. R., Reitsma J. B., de Jongh A., Olff M. (2012). Brief eclectic psychotherapy v. eye movement desensitisation and reprocessing therapy for post-traumatic stress disorder: Randomised controlled trial. *British Journal of Psychiatry*.

[CIT0029] Oren E., Solomon R. (2012). EMDR therapy: An overview of its development and mechanisms of action. *European Review of Applied Psychology*.

[CIT0030] Raïsanen S., Lehto S. M., Nielsen H. S., Gissler M., Kramer M. R., Heinonen S. (2014). Fear of childbirth in nulliparous and multiparous women: A population-based analysis of all singleton births in Finland in 1997-2010. *British Journal of Obstetrics and Gynaecology*.

[CIT0031] Rouhe H., Salmela-Aro K., Halmesmäki E., Saisto T. (2009). Fear of childbirth according to parity, gestational age, and obstetric history. *British Journal of Obstetrics and Gynaecology*.

[CIT0032] Rouhe H., Salmela-Aro K., Toivanen R., Tokola M., Halmesmäki E., Ryding E. L., Saisto R. (2015a). Group psychoeducation with relaxation for severe fear of childbirth improves maternal adjustment and childbirth experience – a randomised controlled trial. *Journal of Psychosomatic Obstetrics and Gynaecology*.

[CIT0033] Rouhe H., Salmela-Aro K., Toivanen R., Tokola M., Halmesmäki E., Saisto T. (2013). Obstetric outcome after intervention for severe fear of childbirth in nulliparous women - Randomised trial. *British Journal of Obstetrics and Gynaecology*.

[CIT0034] Rouhe H., Salmela-Aro K., Toivanen R., Tokola M., Halmesmäki E., Saisto T. (2015b). Life satisfaction, general well-being and costs of treatment for severe fear of childbirth in nulliparous women by psychoeducative group or conventional care attendance. *Acta Obstetricia et Gynecologica Scandinavica*.

[CIT0035] Ryding E. L., Persson A., Onell C., Kvist L. (2003). An evaluation of midwives’ counseling of pregnant women in fear of childbirth. *Acta*.

[CIT0036] Ryding E. L., Wijma B., Wijma K., Rydhström H. (1998). Fear of childbirth during pregnancy may increase the risk of emergency cesarean section. *Acta Obstetricia et Gynecologica Scandinavica*.

[CIT0037] Saisto T., Salmela-Aro K., Nurmi J. E., Könönen T., Halmesmäki E. (2001). A randomized controlled trial of intervention in fear of childbirth. *Obstetrics and Gynecology*.

[CIT0038] Sandström M., Wiberg B., Wikman M., Willman A. K., Högberg U. (2008). A pilot study of eye movement desensitisation and reprocessing treatment (EMDR) for post-traumatic stress after childbirth. *Midwifery*.

[CIT0039] Seng J. S., Low L., Sperlich M., Ronis D., Liberzon I. (2011). Post-traumatic stress disorder, child abuse history, birthweight and gestational age: A prospective cohort study. *BJOG*.

[CIT0040] Seng J. S., Oakley D. J., Sampselle C., Killion C., Graham-Bermann S., Liberzon I. (2001). Posttraumatic stress disorder and pregnancy complications. *Obstetrics and Gynecology*.

[CIT0041] Shaw J., Asch S., Kimderlin R., Frayne S., Shaw K., Phibbs C. (2014). Posttraumatic stress disorder and risk of sponteaneous preterm birth. *Obstetrics and Gynecology*.

[CIT0042] Sheehan D. V., Lecrubier Y., Sheehan K. H., Amorim P., Janavs J., Weiller E., Dunbar G. (1998). The Mini-International Neuropsychiatric Interview (M.I.N.I.): The development and validation of a structured diagnostic psychiatric interview for DSM-IV and ICD-10. *The Journal of Clinical Psychiatry*.

[CIT0043] Söderquist J., Wijma B., Thorbert G., Wijma K. (2009). Risk factors in pregnancy for post-traumatic stress and depression after childbirth. *British Journal of Obstetrics and Gynaecology*.

[CIT0044] Söderquist J., Wijma B., Wijma K. (2006). The longitudinal course of post-traumatic stress after childbirth. *Journal of Psychosomatic Obstetrics and Gynaecology*.

[CIT0045] Spinhoven P., Ormel J., Sloekers P., Kempen G., Speckens A., Van Hemert A. (1997). A validation study of the Hospital anxiety and depression scale (HADS) in different groups of Dutch subjects. *Psychological Medicine*.

[CIT0046] Størksen H. T., Garthus-Niegel S., Adams S. S., Vangen S., Eberhard-Gran M. (2015). Fear of childbirth and elective caesarean section: A population-based study. *BMC Pregnancy and Childbirth*.

[CIT0047] Størksen H. T., Garthus-Niegel S., Vangen S., Eberhard-Gran M. (2013). The impact of previous birth experiences on maternal fear of childbirth. *Acta Obstetricia et Gynecologica Scandinavica*.

[CIT0048] Stramrood C. A. I., Van der Velde J., Doornbos B., Paarlberg K. M., Weijmar Schultz W. C., Van Pampus M. G. (2012). The patient observer: Eye-movement desensitization and reprocessing for the treatment of posttraumatic stress following childbirth. *Birth*.

[CIT0049] Stramrood C. A. I., Wessel I., Doornbos B., Aarnoudse J. G., Van den Berg P. P., Schultz W. C., Van Pampus M. G. (2011). Posttraumatic stress disorder following preeclampsia and PPROM: A prospective study with 15 months follow-up. *Reproductive Sciences*.

[CIT0050] Toohill J., Fenwick J., Gamble J., Creedy D. K., Buist A., Turkstra E., Ryding E. L. (2014). A randomized controlled trial of a psycho-education intervention by midwives in reducing childbirth fear in pregnant women. *Birth*.

[CIT0051] Vesga-López O., Blanco C., Keyes K., Ofson M., Grant B. F., Hasin D. S. (2008). Psychiatric disorders in pregnant and postpartum women in the United States. *Archives of General Psychiatry*.

[CIT0052] Weathers F. W., Blake D. D., Schnurr P. P., Kaloupek D. G., Marx B. P., Keane T. (2013). The life events checklist for DSM-5 (LEC-5). http://www.ptsd.va.gov.

[CIT0053] Weathers F. W., Litz B. T., Keane T. M., Palmieri P. A., Marx B. P., Schnurr P. P. (2013). The PTSD checklist for DSM-5 (PCL-5). http://www.ptsd.va.gov.

[CIT0054] WHO (2013). *Guidelines for the management of conditions that are specifically related to stress*.

[CIT0055] Wijma K., Wijma B., Zar M. (1998). Psychometric aspects of the W-DEQ; a new questionnaire for the measurement of fear of childbirth. *Journal of Psychosomatic Obstetrics and Gynaecology*.

[CIT0056] Wolitzky-Taylor K. B., Horowitz J. D., Powers M. B., Telch M. J. (2008). Psychological approaches in the treatment of specific phobias: A meta-analysis. *Clinical Psychology Review*.

[CIT0057] Yonkers K. A., Smith M. V., Forray A., Epperson C. N., Costello D., Lin H., Belanger K. (2014). Pregnant women with posttraumatic stress disorder and risk of preterm birth. *JAMA Psychiatry*.

[CIT0058] Zigmond A. S., Snaith R. P. (1983). The hospital anxiety and depression scale. *Acta Psychiatrica Scandinavica*.

