# Supplementary material for: The OptiMUM-study: EMDR therapy in pregnant women with posttraumatic stress disorder after previous childbirth and pregnant women with fear of childbirth: design of a multicentre randomized controlled trial
Source: Eur J Psychotraumatol. 2017 Feb 24;8(1):1293315. doi: 10.1080/20008198.2017.1293315 (PMC5345578; doi:10.1080/20008198.2017.1293315)
Supplement: Dutch Extended Abstract [file zept_a_1293315_sm5489.pdf]

EXTENDED ABSTRACT (DUTCH)

De OptiMUM-studie: EMDR therapie bij zwangeren met een posttraumatische stress-stoornis na een vorige bevalling, en zwangeren met bevalangst: een multicenter gerandomiseerd onderzoek met controlegroep (RCT).

Inleiding: circa 3% van de vrouwen ontwikkelt een posttraumatische stress-stoornis (PTSS) na de bevalling, en 7,5% van de zwangeren heeft een pathologische bevalangst (Fear of Childbirth, FoC). Bevalangst en bevallings-gerelateerde PTSS kunnen tijdens de (volgende) zwangerschap leiden tot negatieve maternale en neonatale uitkomsten, en het verzoek om een sectio caesarea.
Eye Movement Desensitization and Reprocessing (EMDR) therapie is een bewezen effectieve behandeling voor PTSS in het algemeen en voor verschillende subtypes van specifieke fobie. Er is weinig literatuur beschikbaar bekend betreffende het toepassen van EMDR therapie tijdens de zwangerschap.

Doel: het doel van deze studie is vaststellen of EMDR therapie een effectieve en veilige behandeling is voor zwangeren met bevalangst of bevallings-gerelateerde PTSS. Daarnaast wordt een kosten-effectenanalyse uitgevoerd.

Methode: De eenzijdig-geblindeerde OptiMUM-studie bestaat uit twee twee-armige gerandomiseerde onderzoeken met controlegroep (RCT), met een overlappend ontwerp. In meerdere ziekenhuizen en eerstelijns verloskundigenpraktijken in Amsterdam, Nederland, wordt aan vrouwen met een amenorroeduur tussen circa 8 en 20 weken gevraagd de Wijma Delivery Expectations Questionnaire (WDEQ) in te vullen om bevalangst vast te stellen. Aan vrouwen die eerder bevallen zijn wordt tevens gevraagd de PTSD Checklist for DSM-5 (PCL-5) in te vullen om te screenen op PTSS na een vorige bevalling. Indien er sprake is van bevalangst of er boven de afkapwaarde van de PCL-5 wordt gescoord volgt er een klinisch interview. In het klinisch interview wordt met behulp van de Mini International Neuropsychiatric Interview- Plus (MINI-PLUS) comorbiditeit vastgesteld, en met de Clinician Administered PTSD Scale (CAPS-5) kan een PTSS worden gediagnosticeerd.
Vijftig vrouwen met bevallings-gerelateerde PTSS en 120 vrouwen met bevalangst worden met behulp van een computerprogramma gerandomiseerd tussen EMDR therapie of standaard zwangerschapsbegeleiding. De EMDR therapie bestaat uit drie sessies bij een

eerstelijnspsycholoog, waarbij volgens protocol gefocust wordt op de meest stressvolle beelden van de traumatische gebeurtenis, of het 'worst-case scenario' bij bevalangst, waarna een serie van oogbewegingen als bilaterale stimulatie wordt uitgevoerd. De standaard zwangerschapsbegeleiding bestaat uit routine antenatale zorg, waarbij elke vorm van extra zorg (aangeboden of op eigen initiatief gezocht) zal worden geregistreerd.
Exclusiecriteria zijn een leeftijd onder de 18 jaar, huidige psychologische behandeling, verhoogd suïciderisico en een ernstige psychotische stoornis. Primaire uitkomstmaten zijn ernst van de symptomen van de bevallings-gerelateerde PTSS of bevalangst. Secundaire uitkomstmaten zijn het percentage PTSS-diagnoses, percentage sectio caesarea, subjectieve bevallingservaring, obstetrische en neonatale complicaties, en kosten. De studie is goedgekeurd door de Medical research Ethics Committees United, en is geregistreerd in het Nederlands Trialregister.

Discussie: De OptiMUM-studie is het eerste gerandomiseerde onderzoek met controlegroep (RCT) voor EMDR therapie tijdens de zwangerschap bij vrouwen met bevallings-gerelateerde PTSS of bevalangst.

Hoogtepunten van het artikel:

- 7,5% van de zwangeren heeft een pathologische bevalangst; 3% van alle vrouwen ontwikkelt een posttraumatische stress-stoornis na de bevalling. Bij deze groepen kan de (volgende) zwangerschap gepaard gaan met hevige psychologische stress en een verhoogd risico op maternale en neonatale complicaties.
- De OptiMUM-studie is het eerste gerandomiseerde onderzoek met controlegroep. (RCT) aangaande EMDR therapie voor zwangeren met PTSS of bevalangst.
- In dit onderzoek worden naast effectiviteit en veiligheid, ook obstetrische en neonatale uitkomstmaten geanalyseerd.
